# Mazdutide Ameliorates Metabolic Dysfunction-Associated Steatotic Liver Disease by Modulating Endoplasmic Reticulum Stress, Improving Lipid Metabolism and Alleviating Inflammation

**DOI:** 10.3390/ph19030371

**Published:** 2026-02-26

**Authors:** Liangyu Gan, Lengxin Duan, Xueyi Zheng

**Affiliations:** 1 College of Basic Medicine and Forensic Medicine, Henan University of Science and Technology, Luoyang 471023, China; g1351396@163.com (L.G.); zxyc0314@163.com (X.Z.); 2 School of Life and Health Sciences, Huzhou College, Huzhou 313000, China

**Keywords:** Mazdutide, MASLD, GLP-1, ER stress, PERK pathway, lipid metabolism, NF-κB

## Abstract

**Background:** Metabolic Dysfunction-Associated Steatotic Liver Disease (MASLD) is the most prevalent chronic liver disorder globally. Mazdutide has shown clinical benefits in weight management and metabolic regulation, indicating its potential as a therapeutic agent for MASLD. This study aimed to investigate the efficacy and mechanism of action of Mazdutide against early-stage MASLD. **Methods:** A MASLD mouse model was induced by a 12-week high-fat diet, followed by a 4-week treatment with subcutaneous Mazdutide (100, 200, or 400 μg/kg). In vitro, a cellular MASLD model was established by treating hepatocytes with 1 mM free fatty acids for 24 h, followed by co-treatment with Mazdutide (10, 20, or 50 nM) or the endoplasmic reticulum (ER) stress inhibitor 4-phenylbutyric acid (4-PBA). Serum and hepatic lipid profiles, liver injury markers, and pro-inflammatory cytokines were quantified. Liver histopathology was assessed by hematoxylin and eosin and Oil Red O staining. Protein expression related to ER stress, inflammation, and lipid metabolism was analyzed by immunohistochemistry and Western blot. **Results:** Compared with the MASLD model group, Mazdutide treatment significantly ameliorated systemic and hepatic lipid metabolism disorders, reduced liver injury markers and hepatic steatosis, and mitigated inflammation and oxidative stress in MASLD mice and hepatocytes (*p* < 0.05). Mechanistically, Mazdutide alleviated ER stress by modulating the protein kinase R-like endoplasmic reticulum kinase (PERK) pathway, suppressed the nuclear Factor kappa B (NF-κB)-mediated inflammatory response, and downregulated the expression of key lipogenic regulators including sterol regulatory element-binding protein 1 (SREBP-1), CCAAT/enhancer-binding protein beta (C/EBPβ), and peroxisome proliferator-activated receptor gamma (PPARγ) in both models (*p* < 0.05). **Conclusions:** Our findings demonstrate that Mazdutide alleviates hepatic ER stress in MASLD, suppresses inflammatory responses and improved lipid metabolism, which ultimately attenuates disease progression.

## 1. Introduction

Metabolic Dysfunction-Associated Steatotic Liver Disease (MASLD), formerly known as non-alcoholic fatty liver disease (NAFLD), is a chronic liver disease closely associated with metabolic dysfunction, with a global prevalence of approximately 25% [1], making it the most common chronic liver disease worldwide [2]. This terminology update was proposed to more accurately reflect the central role of metabolic dysfunction in the pathogenesis of the disease [3,4]. The MASLD spectrum progresses from simple hepatic steatosis to metabolic dysfunction-associated steatohepatitis (MASH), and further to liver fibrosis, cirrhosis, and even hepatocellular carcinoma [5,6]. Notably, MASLD not only increases the risk of liver-related diseases but is also associated with cardiovascular disease, chronic kidney disease, and certain extrahepatic cancers [7]. Consequently, MASLD has emerged as a major global health issue, imposing a significant socioeconomic burden [3].

In MASLD, excessive lipid accumulation within hepatocytes leads to lipotoxicity, which disrupts endoplasmic reticulum (ER) homeostasis and triggers ER stress. This stress, through sustained activation of the unfolded protein response (UPR), causes dysregulation of hepatic lipid metabolism and exacerbates hepatic fat accumulation [8]. ER stress activates pro-inflammatory signaling and apoptotic pathways through the UPR pathway, suppresses the expression of antioxidant enzymes, and exacerbates hepatic inflammation and oxidative stress [9,10]. The protein kinase R-like endoplasmic reticulum kinase (PERK) signaling pathway is one of the three core pathways of the UPR. In MASLD, excessive lipid accumulation activates PERK, leading to phosphorylation of its downstream signaling molecule eukaryotic initiation factor 2 alpha (eIF2α) and subsequent upregulation of activating transcription factor 4 (ATF4) and C/EBP homologous protein (CHOP) expression. Studies indicate that inhibiting the PERK pathway not only reduces ER stress but also modulates lipid metabolism, alleviates inflammation and oxidative stress [11,12,13], thereby multidimensionally interfering with the pathological progression of MASLD.

Currently, approved drugs for MASLD are limited, necessitating a deeper understanding of disease mechanisms to develop effective therapies. Mazdutide is indicated for the treatment of obesity and type 2 diabetes mellitus (T2DM), demonstrating clinical benefits in weight management and metabolic regulation. Notably, obesity and T2DM are the most prevalent and critical risk factors for MASLD: the global prevalence of MASLD exceeds 70% in obese populations and approximately 50–70% in T2DM patients, and these three diseases share the core pathological mechanisms of insulin resistance, glycolipid metabolic disorder and chronic low-grade inflammation [14,15]. Unlike existing single-target Glucagon-like peptide-1 (GLP-1) receptor agonists such as semaglutide, Mazdutide exerts synergistic metabolic regulation through dual receptor activation: GLP-1 receptor activation enhances insulin sensitivity, suppresses appetite, improves systemic glucose and lipid metabolism, and reduces free fatty acids and de novo lipogenesis in the liver. Glucagon (GCG) receptor activation directly modulates hepatic metabolism by promoting hepatic gluconeogenesis and accelerating lipolysis in adipose tissue, thereby reducing hepatic lipid accumulation directly [16,17]. Mazdutide’s dual-target pharmacology confers superior efficacy compared to single-target GLP-1 agonists in weight reduction, insulin resistance improvement, and lipid metabolism normalization [18,19,20]. Its mechanism directly addresses the pathophysiological mechanisms of MASLD, positioning it as a more rational and promising therapeutic candidate for this disease.

Beyond its well-validated efficacy in weight management and glycemic control, Mazdutide is currently under active clinical evaluation for the treatment of MASLD [21]—a Phase II clinical trial (NCT06937749) is investigating its efficacy and safety in patients with MASH, and a Phase III clinical trial (NCT06884293) is focused on com-paring the efficacy and safety of IBI362 and semaglutide in patients with MASLD among overweight or obese adults in China. However, the specific therapeutic effects of Mazdutide on MASLD and its underlying molecular mechanisms associated with its metabolic regulatory properties remain largely unelucidated, particularly whether its hepatoprotective effects are achieved by modulating endoplasmic reticulum stress pathways and improving lipid metabolism and alleviating inflammation. This study aims to preliminarily elucidate the therapeutic effects and potential mechanisms of Mazdutide on MASLD through in vitro and in vivo experiments, so as to provide an experimental basis and theoretical support for the clinical application of Mazdutide and similar drugs in the treatment of MASLD.

## 2. Results

### 2.1. Effects of Mazdutide on Body Weight, Liver-to-Body Weight Ratio, and Liver Morphology in MASLD Mice

**Figure 1:**
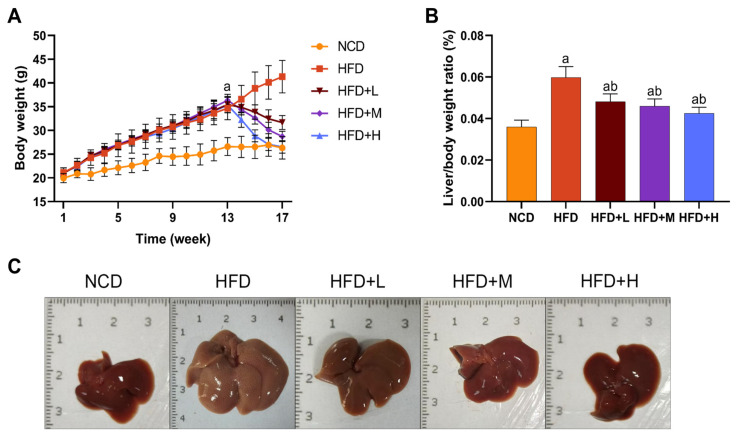
Effects of Mazdutide on body weight, liver-to-body weight ratio, and liver morphology in MASLD mice. (**A**) Dynamic changes in body weight from the beginning of the acclimation period to the end of the experiment. (**B**) Quantification of the liver-to-body weight ratio. (**C**) Representative macroscopic photographs of liver specimens from each group. Data are presented as the mean ± standard deviation (*n* = 15). ^a^ *p* < 0.01 vs. the NCD group; ^b^ *p* < 0.01 vs. the HFD group. Abbreviations: NCD, normal chow diet; HFD, high-fat diet; HFD+L/M/H, HFD + low/medium/high-dose Mazdutide (100/200/400 μg/kg, every 3 days, s.c.).

The effects of Mazdutide on MASLD progression were assessed by monitoring weekly body weight, terminal liver-to-body weight ratio, and liver morphology. After a 1-week acclimation, initial body weights were comparable across all groups. After 13 weeks, mice on a high-fat diet (HFD) exhibited significantly higher body weight gain compared to those on a normal chow diet (NCD) (Figure 1A). Following 4 weeks of drug treatment, the liver-to-body weight ratio was markedly elevated in the HFD group compared with the NCD group, and this increase was significantly reversed by Mazdutide treatment at all doses compared with the HFD group (Figure 1B). Macroscopically, livers from the NCD group displayed a normal deep-red color and texture. In stark contrast, HFD-fed mice developed pale yellow, enlarged, and friable livers with a granular surface, indicative of severe steatosis. Mazdutide treatment improved these gross morphological alterations (Figure 1C).

### 2.2. Effects of Mazdutide on Serum Lipids and Liver Enzymes in MASLD Mice

**Figure 2:**
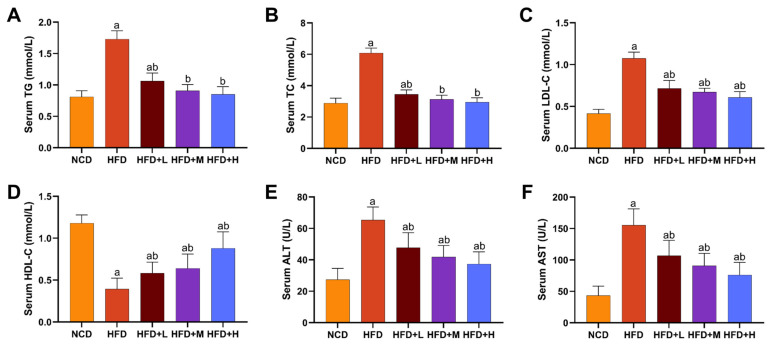
Effects of Mazdutide on serum lipids and liver enzymes in MASLD mice. (**A**–**D**) Serum evels of triglycerides (TG), total cholesterol (TC), low-density lipoprotein cholesterol (LDL-C), and high-density lipoprotein cholesterol (HDL-C). (**E**,**F**) Serum activities of alanine aminotransferase (ALT) and aspartate aminotransferase (AST). Data are presented as the mean ± SD (*n* = 10–15 per group). ^a^ *p* < 0.05 vs. the NCD group; ^b^ *p* < 0.05 vs. the HFD group. Animal groups are defined in Figure 1.

Mazdutide’s effects on lipid metabolism and hepatoprotection were evaluated by measuring serum lipid profiles and liver enzyme activities. The HFD group displayed a dyslipidemic profile, characterized by significantly elevated levels of total cholesterol (TC), triglycerides (TG), and low-density lipoprotein cholesterol (LDL-C), alongside reduced high-density lipoprotein cholesterol (HDL-C), compared to the NCD group. The HFD+L/M/H group significantly reversed these HFD-induced alterations, lowering TC, TG, and LDL-C and increasing HDL-C relative to the HFD group (Figure 2A–D). Serum TG and TC levels in the HFD+M and HFD+H groups returned to the levels of the NCD group (Figure 2A,B). Consistently, serum alanine aminotransferase (ALT) and aspartate aminotransferase (AST) levels were markedly increased in the HFD group compared to the NCD group, indicating hepatic injury. All doses of Mazdutide treatment significantly reduced ALT and AST levels compared to the HFD group (Figure 2E,F).

### 2.3. Mazdutide Attenuates Hepatic Steatosis and Pathological Injury in MASLD Mice

**Figure 3:**
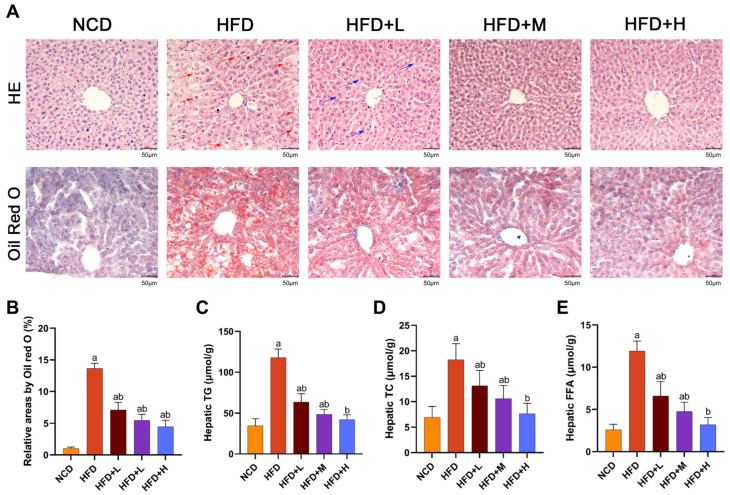
Mazdutide attenuates hepatic steatosis and pathological injury in MASLD mice. (**A**) Representative photomicrographs of liver sections stained with hematoxylin and eosin (HE) and Oil Red O (scale bar = 50 μm; 200× magnification, *n* = 5). Red arrows indicate hepatic steatosis, ballooning degeneration, and hepatocyte edema; blue arrows indicate mild hepatocyte edema. (**B**) Quantification of hepatic lipid droplets by Oil Red O staining (*n* = 5). (**C**–**E**) Hepatic levels of TG, TC, and free fatty acid (FFA) (*n* = 10–15). Data are presented as the mean ± SD. ^a^ *p* < 0.01 vs. the NCD group; ^b^ *p* < 0.01 vs. the HFD group. Animal groups are defined in Figure 1.

Histopathology and hepatic lipid accumulation were assessed. Histological analysis by Hematoxylin and Eosin (HE) staining showed normal liver architecture in the NCD group. The HFD group exhibited disorganized hepatocyte arrangement, hepatic steatosis, ballooning degeneration, and hepatocyte edema. Compared with the HFD group, Mazdutide treatment significantly alleviated these pathological changes. Specifically, the HFD+L group showed only mild hepatocyte edema, while the hepatic morphology in the HFD+M and HFD+H groups was close to that of the NCD group. Oil Red O staining consistently revealed markedly increased lipid droplet accumulation in the HFD group compared with the NCD group. Treatment with Mazdutide significantly reduced the Oil Red O-stained area of lipid droplets compared with the HFD group (Figure 3A,B). Liver tissue levels of TG, TC, and free fatty acid (FFA) were significantly higher in the HFD group than in the NCD group. Compared with the HFD group, lipid levels were reduced in the HFD+L/M/H group. There was no significant difference in hepatic TG, TC, and FFA levels between the HFD+H group and the NCD group, this indicates that TG, TC, and FFA levels in the liver of the HFD+H group returned to normal (Figure 3C–E).

### 2.4. Mazdutide Alleviates Inflammation and Hepatic Oxidative Stress in MASLD Mice

**Figure 4:**
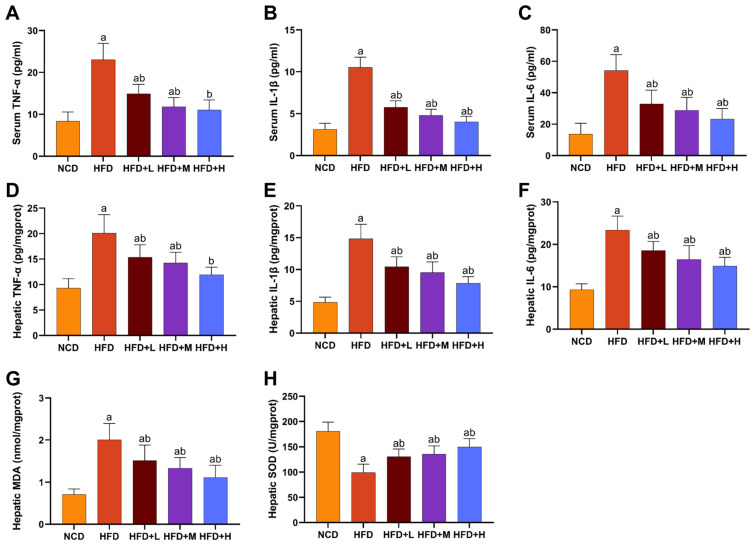
Mazdutide alleviates inflammation and hepatic oxidative stress in MASLD mice. (**A**–**C**) Serum concentrations of tumor necrosis factor-α (TNF-α), interleukin-1β (IL-1β), and interleukin-6 (IL-6). (**D**–**F**) Hepatic levels of TNF-α, IL-1β, and IL-6. (**G**,**H**) Hepatic levels of malondialdehyde (MDA) and superoxide dismutase (SOD) activity. Data are presented as the mean ± SD (*n* = 10–15 per group). ^a^ *p* < 0.01 vs. the NCD group; ^b^ *p* < 0.01 vs. the HFD group. Animal groups are defined in Figure 1.

Serum and hepatic levels of the pro-inflammatory cytokines tumor necrosis factor-alpha (TNF-α), interleukin-6 (IL-6), and interleukin-1 beta (IL-1β) were significantly elevated in the HFD group compared to the NCD group. Mazdutide treatment significantly reduced the levels of all three cytokines compared with the HFD group (Figure 4A–F). Consistently, hepatic oxidative stress was markedly induced by HFD, as evidenced by increased malondialdehyde (MDA) content and decreased superoxide dismutase (SOD) activity compared with the NCD group. Mazdutide administration reversed these alterations, significantly lowering MDA levels and enhancing SOD activity compared to the HFD group (Figure 4G,H).

### 2.5. Mazdutide Suppresses ER Stress via the PERK Pathway in MASLD Mouse Liver Tissue

**Figure 5:**
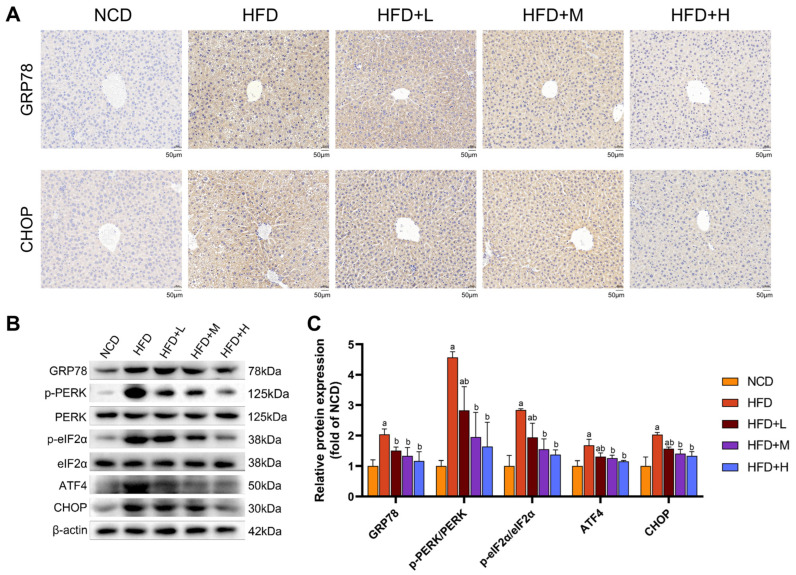
Mazdutide suppresses ER stress via the PERK pathway in MASLD mouse liver tissue. (**A**) Representative immunohistochemical staining of GRP78 and CHOP in liver sections (scale bar = 50 μm, 200× magnification). (**B**) Representative Western blot images of key proteins in the PERK pathway from each experimental group. (**C**) Quantitative densitometric analysis of PERK and eIF2α phosphorylation (presented as the p-PERK/PERK and p-eIF2α/eIF2α ratios) and the protein levels of GRP78, ATF4, and CHOP. All values were normalized to β-actin. Data are presented as the mean ± SD (*n* = 5). ^a^ *p* < 0.05 vs. the NCD group; ^b^ *p* < 0.05 vs. the HFD group. Animal groups are defined in Figure 1.

To assess ER stress, we analyzed key markers by immunohistochemistry and Western blot. Immunohistochemistry revealed strong positive staining for GRP78 and CHOP in the HFD group, which was visibly attenuated in all Mazdutide-treated groups compared to the HFD group (Figure 5A). Western blot analysis confirmed these findings. Protein levels of the ER chaperone GRP78 were significantly elevated in the HFD group compared to the NCD group. Furthermore, key components of the PERK-eIF2α-ATF4-CHOP branch were markedly activated in the HFD group, as evidenced by increased phosphorylation levels of PERK (p-PERK/PERK ratio) and eIF2α (p-eIF2α/eIF2α ratio), along with upregulated protein expression of ATF4 and its downstream target CHOP compared with the NCD group. Mazdutide treatment reversed these HFD-induced increases, significantly reducing the phosphorylation of PERK and eIF2α and downregulating the protein levels of GRP78, ATF4, and CHOP compared to the HFD group (Figure 5B,C).

### 2.6. Mazdutide Downregulates Hepatic Inflammation and Lipogenesis in a Mouse Model of MASLD

**Figure 6:**
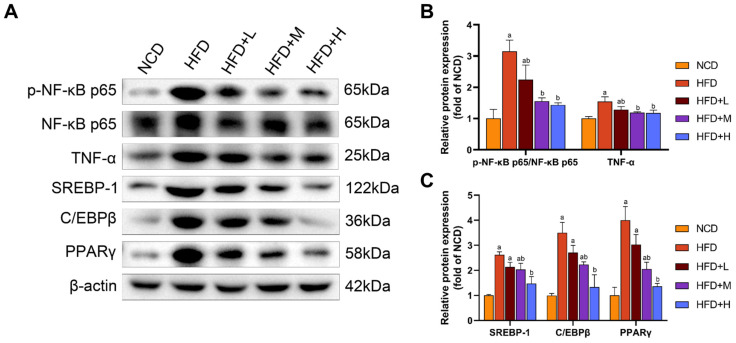
Mazdutide downregulates hepatic inflammation and lipogenesis in a mouse model of MASLD. (**A**) Representative Western blot images of phosphorylated nuclear factor kappa-B p65 (p-NF-κB p65), total NF-κB p65, TNF-α, sterol regulatory element-binding protein 1 (SREBP-1), CCAAT/enhancer-binding protein beta (C/EBPβ), peroxisome proliferator-activated receptor gamma (PPARγ), and β-actin. (**B**) Quantitative densitometric analysis of the p-NF-κB p65 to NF-κB p65 ratio and TNF-α protein levels. (**C**) Quantitative densitometric analysis of SREBP-1, C/EBPβ, and PPARγ protein levels. All protein levels were normalized to β-actin. Data are presented as the mean ± SD (*n* = 5). ^a^ *p* < 0.05 vs. the NCD group; ^b^ *p* < 0.05 vs. the HFD group. Animal groups are defined in Figure 1.

Hepatic expression of key inflammatory and lipogenic proteins was assessed by Western blot (Figure 6). Compared to the NCD group, the HFD group showed a significant increase in the ratio of phosphorylated nuclear factor kappa-B p65 (p-NF-κB p65) to total NF-κB p65 (p-NF-κB p65/NF-κB p65) and the protein level of TNF-α, indicating activation of the NF-κB pathway. Concurrently, the expression of lipogenic transcription factors—sterol regulatory element-binding protein 1 (SREBP-1), CCAAT/enhancer-binding protein beta (C/EBPβ), and peroxisome proliferator-activated receptor gamma (PPARγ)—was also significantly elevated in the HFD group. Treatment with Mazdutide significantly reversed these HFD-induced changes. Compared to the HFD group, the p-NF-κB p65/NF-κB p65 ratio and the expression levels of TNF-α, SREBP-1, C/EBPβ, and PPARγ were all significantly downregulated in the liver.

### 2.7. Mazdutide Reduces Lipid Accumulation in an MASLD Cell Model and Informs Concentration Selection

**Figure 7:**
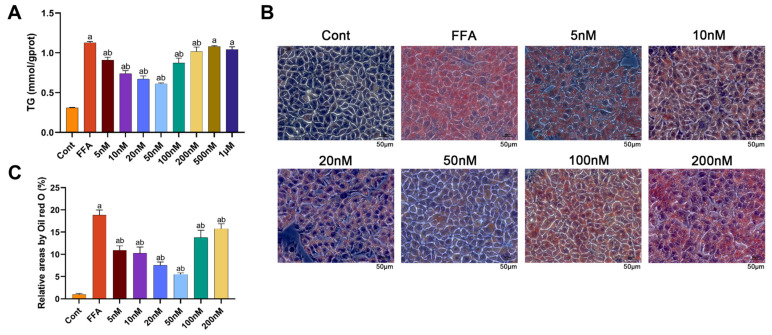
Mazdutide reduces lipid accumulation in an MASLD cell model and informs concentration selection. (**A**) Intracellular TG content in cells treated with 1 mM FFA and increasing concentrations of Mazdutide (5 nM to 1 μM) for 24 h. (**B**) Representative photomicrographs of Oil Red O staining showing intracellular lipid droplets (scale bar = 50 μm, 400× magnification). (**C**) Quantification of cellular lipid droplets by Oil Red O staining. Data are presented as the mean ± SD, *n* = 3 independent experiments. ^a^ *p* < 0.01 vs. the NCD group; ^b^ *p* < 0.05 vs. the HFD group. Group definitions: Cont, control (BSA-containing medium); FFA, 1 mM free fatty acids; All other groups were co-treated with 1 mM FFA plus the indicated concentrations of Mazdutide.

An in vitro MASLD model was established in Alpha Mouse Liver-12 (AML-12) cells using 1 mM FFA (FFA group), and the control group (Cont) was treated with medium containing the same concentration of bovine serum albumin (BSA) solvent as the FFA group. Cells were co-treated with 1 mM FFA and varying concentrations of Mazdutide (5 nM–1 μM). Compared to the Cont group, intracellular TG levels were significantly increased by FFA, but were markedly reduced by Mazdutide at concentrations ranging from 5 nM to 200 nM (Figure 7A), with the most pronounced reductions observed at 10, 20, and 50 nM. Similarly, Oil Red O staining demonstrated that lipid droplet accumulation induced by FFA was visibly diminished by Mazdutide at 5 nM to 200 nM compared to the FFA group (Figure 7B,C), most notably at 10, 20, and 50 nM. The 50 nM treatment restored cell morphology most closely to that of the Cont group. Based on these results, 10 nM (low), 20 nM (medium), and 50 nM (high) Mazdutide were selected for subsequent experiments.

### 2.8. Mazdutide Mitigates Inflammation and Oxidative Stress in MASLD Cells

**Figure 8:**
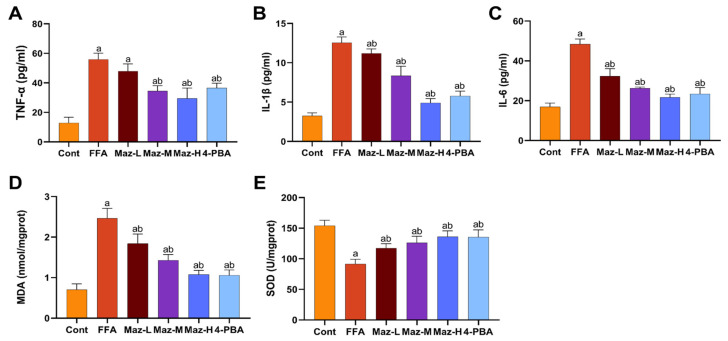
Mazdutide mitigates inflammation and oxidative stress in MASLD cells. (**A**–**C**) Levels of TNF-α, IL-1β, and IL-6 in cell supernatants. (**D**,**E**) Levels of MDA and SOD activity in cell lysates. Data are presented as the mean ± SD (*n* = 3–6). ^a^ *p* < 0.05 vs. the NCD group; ^b^ *p* < 0.05 vs. the HFD group. Group definitions: Cont, control (BSA-containing medium); FFA, 1 mM free fatty acids; Maz-L, 10 nM Mazdutide + 1 mM FFA; Maz-M, 20 nM Mazdutide + 1 mM FFA; Maz-H, 50 nM Mazdutide + 1 mM FFA; 4-PBA, 1 mM 4-PBA + 1 mM FFA.

Inflammatory and oxidative stress markers were quantified in the MASLD cell model. Compared to the Cont group, the FFA group exhibited significantly elevated levels of TNF-α, IL-6, and IL-1β, alongside decreased SOD activity and increased MDA content. These FFA-induced alterations were significantly reversed by co-treatment with low-dose Mazdutide (Maz-L), medium-dose Mazdutide (Maz-M), high-dose Mazdutide (Maz-H), or ER stress inhibitor 4-phenylbutyric acid (4-PBA) (Figure 8).

### 2.9. Mazdutide Inhibits the PERK-eIF2α-ATF4-CHOP Pathway in MASLD Cells

**Figure 9:**
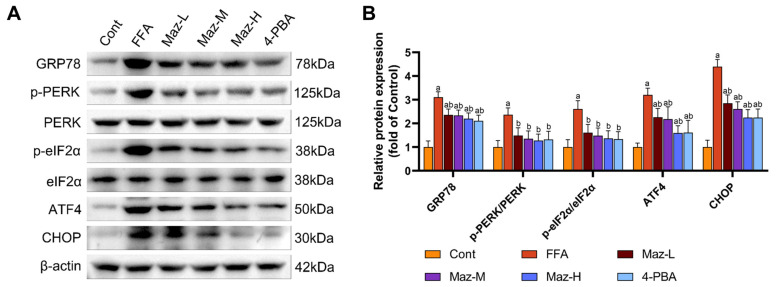
Mazdutide inhibits the PERK-eIF2α-ATF4-CHOP pathway in MASLD cells. (**A**) Representative Western blot images showing the protein levels of GRP78, p-PERK, PERK, p-eIF2α, eIF2α, ATF4, CHOP, and β-actin. (**B**) Quantitative densitometric analysis of the p-PERK/PERK and p-eIF2α/eIF2α ratios and the protein levels of GRP78, ATF4, and CHOP. The protein band intensities were normalized to β-actin. Data are presented as the mean ± SD (*n* = 3). ^a^ *p* < 0.05 vs. the NCD group; ^b^ *p* < 0.05 vs. the HFD group. Cell groups are as defined in Figure 8.

Western blot analysis of key ER stress markers demonstrated that FFA challenge robustly activated the PERK-eIF2α-ATF4-CHOP pathway in hepatocytes. This activation was evidenced by a significant upregulation of GRP78, ATF4, and CHOP, alongside increased phosphorylation of PERK and eIF2α, relative to the Cont group. Notably, co-treatment with Mazdutide (at low, medium, or high doses) or the chemical chaperone 4-PBA effectively mitigated this stress response, with all intervention groups exhibiting markedly attenuated expression of these proteins compared to the FFA model group (Figure 9).

### 2.10. Mazdutide Reduces the Expression of Inflammation and Lipid Metabolism-Related Proteins in MASLD Cells

**Figure 10:**
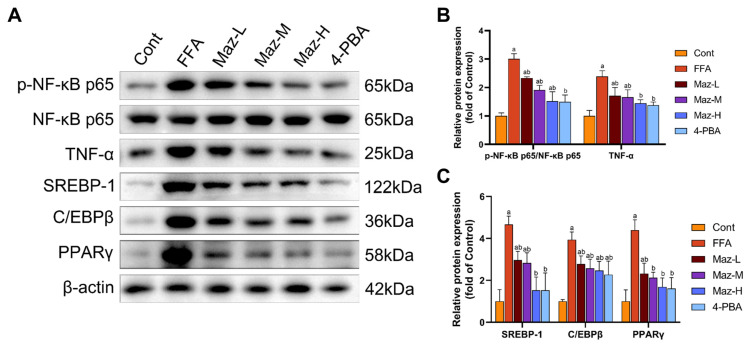
Mazdutide reduces the expression of inflammation and lipid metabolism-related proteins in MASLD cells. (**A**) Representative Western blot images of p-NF-κB p65, NF-κB p65, TNF-α, SREBP-1, C/EBPβ, PPARγ, and β-actin. (**B**) Quantitative densitometric analysis of the p-NF-κB p65 to NF-κB p65 ratio and TNF-α protein levels. (**C**) Quantitative densitometric analysis of SREBP-1, C/EBPβ, and PPARγ protein levels. All protein levels were normalized to β-actin. Data are presented as the mean ± SD (*n* = 3). ^a^ *p* < 0.05 vs. the NCD group; ^b^ *p* < 0.05 vs. the HFD group. Cell groups are as defined in Figure 8.

Western blot analysis revealed that FFA treatment simultaneously activated pro-inflammatory and lipogenic pathways in hepatocytes. Specifically, it significantly enhanced NF-κB pathway activity, evidenced by an increased p-NF-κB p65/NF-κB p65 ratio, and upregulated the expression of the downstream cytokine TNF-α and the key lipogenic regulators SREBP-1, C/EBPβ, and PPARγ, compared to the Cont group. Importantly, co-administration of Mazdutide (at low, medium, or high doses) or the ER stress inhibitor 4-PBA effectively countered these changes, significantly attenuating NF-κB activation and reducing the expression of TNF-α, SREBP-1, C/EBPβ, and PPARγ compared to the FFA model group (Figure 10).

## 3. Discussion

MASLD, formerly known as NAFLD, represents the most prevalent chronic liver disorder globally, posing a significant public health burden. The current lack of targeted pharmacotherapies underscores an urgent need for effective and safe intervention strategies. Mazdutide, a dual GLP-1 and GCG receptor agonist, has shown efficacy in weight reduction and glycemic control. However, its therapeutic potential and precise molecular mechanisms in MASLD remain insufficiently defined [21]. This study utilized an HFD-induced murine MASLD model to replicate common unhealthy dietary patterns. Concurrently, an in vitro MASLD model was established by treating AML-12 cells with FFA at a final concentration of 1 mM, composed of oleic acid (OA) and palmitic acid (PA) in a 2:1 molar ratio, mimicking the imbalanced fatty acid milieu observed in patients. We systematically evaluated the therapeutic effects of Mazdutide on MASLD and explored its underlying mechanisms, thereby providing preclinical evidence to support its clinical translation.

Previous studies [22,23] have demonstrated that the progression of MASLD typically involves hepatic lipid accumulation, hepatocyte injury, and impaired liver function, often accompanied by chronic inflammation and oxidative stress—pathological features all reproduced in our model. Our findings indicate that Mazdutide exerts protective effects against MASLD at both the animal and cellular levels. Mice fed a high-fat diet developed severe dyslipidemia and hepatic dysfunction, which were significantly ameliorated following Mazdutide treatment. Histopathological analysis further revealed that Mazdutide markedly reduced hepatic steatosis and lipid droplet accumulation. Consistent results were observed in both in vivo and in vitro experiments: Mazdutide decreased oxidative stress levels and reduced proinflammatory cytokine secretion in the MASLD model. Collectively, these findings align with previous reports on similar agents (e.g., semaglutide), indicating that GLP-1-based therapies can mitigate MASLD progression [24,25,26]. By demonstrating Mazdutide’s protective effects against MASLD, this study expands existing knowledge and further validates the therapeutic potential of dual GLP-1/GCG agonists for MASLD.

Notably, during in vitro studies investigating Mazdutide’s lipid-lowering effects and determining its optimal concentration range, we observed that Mazdutide significantly reduced TG levels at lower doses (10 nM–50 nM). While at higher doses (≥100 nM), TG levels increased. This suggests Mazdutide may exhibit a “Low concentrations reduce lipid accumulation, while high concentrations diminish the effect” profile at the cellular level. Considering Mazdutide’s pharmacological properties as a dual GLP-1/GCG receptor agonist and established literature patterns, we hypothesize that its potential mechanism may involve dual receptor signaling imbalance: at concentrations exceeding 100 nM, the GLP-1 receptor may undergo phosphorylation-dependent endocytosis due to excessive stimulation, reducing the number of effective receptors on the cell membrane surface and consequently weakening the signal inhibiting lipogenesis [27]. In contrast, β-arrestin recruitment at the GCG receptor is phosphorylation-independent, as previously reported [28]; this regulatory feature may confer greater signaling persistence at high concentrations compared to the GLP-1 receptor. This disparity may cause receptor functional imbalance: the GCG receptor continuously drives lipolysis, producing large amounts of free fatty acids, while the GLP-1 receptor-mediated inhibition of lipogenesis is significantly weakened. Fatty acid oxidation capacity reaches saturation, and free fatty acids not promptly oxidized are passively re-esterified into TG and accumulate [29,30]. This ultimately manifests as diminished lipid-lowering effects at high concentrations.

Extensive evidence indicates that ER stress plays a pivotal role in the pathogenesis and progression of MASLD [31,32]. Specifically, excessive lipid accumulation disrupts ER homeostasis, triggering abnormal activation of the UPR. Under stress conditions, expression of the stress sensor GRP78 is upregulated, subsequently activating downstream pathways such as PERK-eIF2α-ATF4-CHOP [8]. Concurrently, chronic ER stress drives lipid synthesis and inflammatory responses, thereby accelerating MASLD progression. This creates a vicious cycle of mutual causation among ER stress, abnormal lipid accumulation, and inflammatory responses within hepatocytes. As previously demonstrated, the lipogenic transcription factor SREBP-1 promotes hepatic lipid accumulation by regulating its downstream lipogenic enzymes [33,34], while ER stress modulates SREBP-1 maturation via the PERK pathway [35,36]. C/EBPβ participates in hepatic metabolic disorders by regulating the expression of lipogenic and inflammation-related genes [37,38]. Its transcriptional activity increases under ER stress, potentially exacerbating lipid abnormalities [39]. PPARγ, a key regulator of lipid storage, is closely associated with hepatic steatosis [40,41]. Under conditions of nutritional excess, PERK activation promotes nuclear translocation of PPARγ [42,43]. Furthermore, PERK-mediated ER stress activates the NF-κB signaling pathway [44], and sustained NF-κB activation may form a vicious cycle exacerbating metabolic dysfunction and ER stress [45,46]. This study found that Mazdutide significantly downregulated GRP78 and key proteins in the PERK-eIF2α-ATF4-CHOP pathway both in vivo and in vitro, thereby alleviating hepatic ER stress. Simultaneously, it downregulated expression of lipid synthesis transcription factors SREBP-1, C/EBPβ, and PPARγ, reduced NF-κB p65 phosphorylation and TNF-α protein expression, thereby mitigating de novo lipogenesis and hepatic inflammation. These multidimensional synergistic regulatory effects resemble those of the classic ER stress inhibitor 4-PBA, supporting a close association between inhibition of the PERK-eIF2α-ATF4-CHOP pathway and improved lipid metabolism alongside reduced inflammation. This suggests that Mazdutide exerts protective effects by alleviating endoplasmic reticulum stress and mitigating associated lipogenesis and inflammatory responses.

In summary, this study confirms Mazdutide’s significant hepatocellular protective effects against MASLD. At the molecular level, Mazdutide regulates the expression of lipogenic and inflammatory molecules and the PERK-eIF2α-ATF4-CHOP signaling pathway, breaking the vicious cycle linking ER stress, abnormal lipid accumulation, and inflammation. This demonstrates its multi-target synergistic protective characteristics. Given the current lack of clinical therapeutic options for MASLD, this study provides preclinical evidence supporting the clinical translation of Mazdutide for this disease and offers novel research perspectives for multi-targeted interventions in metabolic liver diseases.

## 4. Materials and Methods

### 4.1. Reagents

Mazdutide acetate was purchased from Abmole Bioscience Inc. (Houston, TX, USA). TG, TC, LDL-C, HDL-C, ALT, AST, MDA, and SOD assay kits were purchased from Nanjing Jiancheng Bioengineering Institute (Nanjing, China; Catalog Nos.: A110-1-1, A111-1-1, A113-1-1, A112-1-1, C009-2-1, C010-2-1, A003-1, A001-3, respectively). The FFA assay kit was purchased from Abbkine Scientific Co., Ltd. (Wuhan, China; Catalog No.: KTB2230). Mouse TNF-α, mouse IL-1β, and mouse IL-6 ELISA kits were purchased from Jonln Bio (Shanghai, China; Catalog Nos.: JLW10484, JLW18442, JLW20268, respectively). Oleic acid (OA), fatty acid- free and IgG-free bovine serum albumin (BSA), and BCA protein concentration assay kits were purchased from Beyotime Biotechnology (Shanghai, China). Palmitic acid (PA) was purchased from Sigma-Aldrich (St. Louis, MO, USA). 4-PBA was purchased from MedChemExpress (Monmouth Junction, NJ, USA). Primary antibodies for GRP78, ATF4, CHOP, and β-actin were purchased from Proteintech Group, Inc (Wuhan, China). Antibodies for SREBP-1, C/EBPβ, and PPARγ were purchased from ZEN-BIOSCIENCE Co., Ltd. (Chengdu, China). Antibodies for eIF2α and p-eIF2α (Ser51) were purchased from Cell Signaling Technology (Danvers, MA, USA). Antibodies for PERK, p-PERK (T892), NF-κBp65, p-NF-κBp65 (Ser536), and TNF-α were purchased from Wanlei Life Sciences (Shenyang) Co., Ltd. (Shenyang, China). Secondary antibodies were purchased from Beijing Zhongshan Golden Bridge Biotechnology Co., Ltd. (Beijing, China).

### 4.2. Animals and Treatment

Six-week-old male C57BL/6J mice [47] were purchased from Henan Skobes Biotechnology Co., Ltd. (Anyang, China) and housed in a constant temperature (22 ± 2 °C) and humidity (40–60%) environment under a 12 h light/dark cycle. Mice had free access to chow and drinking water. Mice were randomly divided into five groups (*n* = 15 per group). Following a 1-week acclimation, one normal chow diet (NCD) group and four high-fat diet (HFD, 60% kcal from fat) groups were established. The normal chow used in this study was commercially available maintenance chow for mice, purchased from Huanyu Biotechnology Co., Ltd. (Beijing, China). The high-fat diet was custom-produced by Henan Skobes Biotechnology Co., Ltd. (Anyang, China) based on the publicly available D12492 formulation from Research Diets, Inc. (New Brunswick, NJ, USA). For details, the composition of NCD and HFD is shown in Table S1 in the Supplementary Materials.

**Figure 11:**
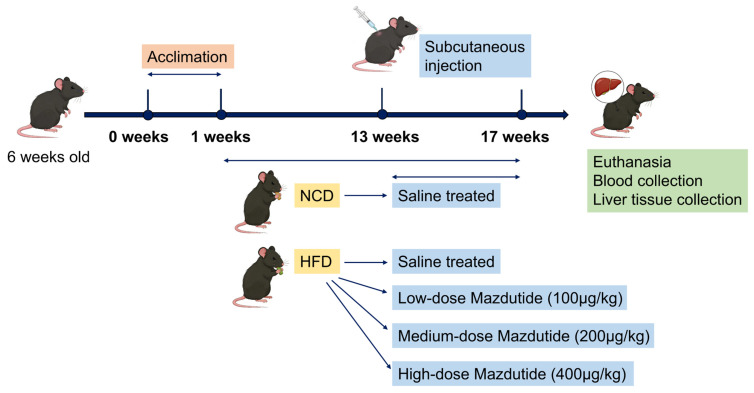
Schematic diagram of the animal experimental design.

Prior to the formal drug administration treatment, a single high dose of Mazdutide (600 μg/kg) was subcutaneously injected into 5 naive healthy SPF male C57BL/6J mice (not included in the formal experimental groups). The general condition, body weight and obvious abnormal reactions were monitored daily for 14 days, with no toxic effects observed in all mice. Based on this safety verification and referring to the effective dose range of Mazdutide in metabolic models [16], 100, 200 and 400 μg/kg were set as the gradient doses for the subsequent formal study. After 12 weeks of HFD feeding, Mazdutide was administered subcutaneously every 3 days for 4 weeks to three HFD-fed groups: the high-fat-diet low-dose group (HFD+L, 100 μg/kg), the high-fat-diet medium-dose group (HFD+M, 200 μg/kg), and the high-fat-diet high-dose group (HFD+H, 400 μg/kg). Mazdutide was dissolved in 0.9% sterile normal saline for subcutaneous injection at respective doses; equal volumes of the same saline were subcutaneously administered to NCD and HFD control mice. At the end of the treatment, all mice were euthanized for the collection of blood and liver samples. A schematic diagram of the animal experiment design was drawn (Figure 11).

### 4.3. Preparation of FFA

PA and OA were individually dissolved in sodium hydroxide (NAOH) solution at a molar ratio of 1:1 (fatty acid:NaOH). Solubilization was facilitated by incubation in a 70 °C water bath, followed by dilution of each fatty acid into 10% fatty-acid-free and IgG-free BSA solution. The resultant PA and OA stock solutions were sterile-filtered through 0.22 μm membranes, aliquoted, and stored at −80 °C in the dark. For experimental use, the working FFA solution was freshly prepared by mixing PA and OA stocks at a molar ratio of 1:2 [48].

### 4.4. Cell Culture and Grouping

AML-12 cell line, an immortalized and non-transformed hepatocyte line, was purchased from Zhongqiaoxinzhou Biotechnology Co., Ltd. (Shanghai, China). AML-12 cells were cultured in DMEM/Ham’s F12 medium supplemented with 10% fetal bovine serum, insulin-transferrin-selenium mixture, 0.1 mM dexamethasone, penicillin, and streptomycin. Cells were cultured at 37 °C in a humidified environment with 5% CO_2_. TG content was measured after co-treating AML-12 cells with varying Mazdutide concentrations (5 nM–1 μM) and 1 mM FFA for 24 h. Lipid droplet formation was visualized via Oil Red O staining. The optimal Mazdutide concentrations for improving lipid accumulation—10 nM, 20 nM, and 50 nM—were selected as low, medium, and high doses, respectively. AML-12 cells were divided into six experimental groups: the Cont group, treated with basal medium supplemented with an equal concentration of BSA as in FFA-treated groups but without FFA; the FFA group, treated with basal medium containing 1 mM free fatty acids to establish a high-fat cell model; three Mazdutide intervention groups co-treated with 1 mM FFA and Mazdutide at low (10 nM), medium (20 nM), or high (50 nM) doses (Maz-L, Maz-M, and Maz-H, respectively); and the 4-PBA group, co-treated with 1 mM FFA and 1 mM 4-PBA. Mazdutide and 4-PBA were first dissolved in basal medium to prepare stock solutions.

### 4.5. Biochemical Parameter Detection

Serum levels of TG, TC, LDL-C, HDL-C, ALT, and AST were measured using commercially available biochemical assay kits according to the manufacturers’ instructions, with TG quantified via the glycerol-3-phosphate oxidase-p-aminophenol (GPO-PAP) enzymatic method (Limit of Detection, LOD: 0.035 mmol/L; Limit of Quantification, LOQ: 0.3 mmol/L), TC via the cholesterol oxidase-p-aminophenol (COD-PAP) method (LOD: 0.28 mmol/L; LOQ: 0.42 mmol/L), LDL-C and HDL-C via a direct enzymatic approach with surfactant-mediated clearance (LOD: 0.075 mmol/L and 0.072 mmol/L; LOQ: 0.61 mmol/L and 0.09 mmol/L, respectively), and ALT and AST via colorimetric detection using 2,4-dinitrophenylhydrazine (LOD: 0.35 U/L and 0.3 U/L; LOQ: 0.62 U/L and 0.74 U/L, respectively). The levels of TG, TC, FFA, MDA, and SOD in 10% (*w*/*v*) mouse liver tissue homogenate were detected according to the kit manufacturer’s instructions, where FFA was assayed by a copper-based microplate colorimetric method (LOD: 0.0156 mM; LOQ: 0.0313 mM), MDA via the thiobarbituric acid reactive substances assay (TBARS) (LOD: 0.36 nmol/mL; LOQ: 0.5 nmol/mL), and SOD activity via the water-soluble tetrazolium salt-1 (WST-1) reduction method (LOD: 2.89 U/mL; LOQ: 5 U/mL). Similarly, the levels of TG, MDA, and SOD in AML-12 cell lysate were detected according to the kit manufacturer’s instructions, utilizing the same reagents and analytical workflows as applied to liver homogenates to maintain assay uniformity.

### 4.6. Inflammatory Cytokine Assay

Inflammatory markers in mouse serum and AML-12 cell culture supernatants, including TNF-α (LOD: 0.59 pg/mL; LOQ: 1.25 pg/mL), IL-1β (LOD: 0.32 pg/mL; LOQ: 0.78 pg/mL), and IL-6 (LOD: 0.74 pg/mL; LOQ: 1.56 pg/mL), were detected using sandwich ELISA kits according to the manufacturers’ instructions. The concentrations of these three inflammatory cytokines in 10% (*w*/*v*) mouse liver tissue homogenates were also quantified via the same protocol.

### 4.7. HE Staining

Liver tissues from corresponding sites in each group of mice were fixed in 4% paraformaldehyde for 24 h, then routinely processed and embedded in paraffin. The paraffin-embedded tissue blocks were sectioned at 5 μm thickness using a rotary microtome. For HE staining, the sections were dewaxed in xylene and rehydrated through a graded ethanol series. Subsequently, the sections were stained with hematoxylin for 10 min, differentiated in 1% acid ethanol for 30 s, and blued in running water for 10 min. This was followed by eosin staining for 5 min. Finally, the stained sections were dehydrated through a graded ethanol series, cleared in xylene, mounted with a neutral resin, and examined under an upright transmitted light microscope (Olympus BX41, Olympus Corporation, Tokyo, Japan) for morphological assessment.

### 4.8. Oil Red O Staining

#### 4.8.1. Tissue Staining

Mouse liver tissues from corresponding sites were embedded in O.C.T. compound, cryopreserved at −80 °C, and sectioned at 10 μm using a cryostat. After fixation with 4% paraformaldehyde and washing, sections were stained with freshly filtered Oil Red O working solution (3:2 stock:water) for 20 min in the dark. Sections were then differentiated in 60% isopropanol, rinsed, and counterstained with hematoxylin. Following bluing in water, sections were mounted with glycerol-gelatin medium and examined by the Olympus BX41 microscope. Lipid droplets within hepatocytes were identified as orange-red deposits.

#### 4.8.2. Cell Staining

AML-12 cells were seeded in 6-well plates. Upon reaching 60–70% confluence, the cells were co-treated with an FFA solution and various concentrations of Mazdutide for 24 h. After treatment, the cells were washed three times with PBS, fixed with 4% paraformaldehyde for 10 min, and then stained using the same Oil Red O staining protocol as described for tissue sections (Section 4.8.1). Finally, the stained cells were washed, and images were acquired using an inverted microscope (Zeiss Axio Observer 3, Carl Zeiss AG, Oberkochen, Germany) in bright-field mode.

### 4.9. Immunohistochemical Staining

Paraffin-embedded mouse liver sections were dewaxed, rehydrated, and subjected to antigen retrieval in citrate buffer (pH 6.0). After blocking endogenous peroxidase with 3% H_2_O_2_ and nonspecific sites with 5% BSA, sections were incubated overnight at 4 °C with primary antibodies against GRP78 or CHOP (1:500). Following incubation with an HRP-conjugated goat anti-rabbit secondary antibody (1:200), immunoreactivity was visualized using a DAB kit. Nuclei were counterstained with hematoxylin. Sections were then dehydrated, cleared, mounted, and examined under an upright light microscope (Nikon ECLIPSE E100, Nikon Corporation, Tokyo, Japan). Positive expression was identified by yellow-brown cytoplasmic or nuclear granules.

### 4.10. Western Blotting

Proteins were extracted from liver tissues and hepatocytes using RIPA buffer with phosphatase and protease inhibitors. Concentrations were determined by BCA assay. Equal protein amounts were denatured, separated by SDS-PAGE, and transferred to PVDF membranes. After blocking with 5% non-fat milk in TBST, membranes were incubated overnight at 4 °C with primary antibodies, followed by HRP-conjugated secondary antibodies for 1 h at room temperature. Following TBST washes, bands were visualized using an enhanced chemiluminescence (ECL) detection system.

### 4.11. Statistical Analysis

Statistical analyses were performed using GraphPad Prism 9.0. Data from ≥3 independent experiments are expressed as mean ± SD. Multiple group comparisons used one-way ANOVA followed by Tukey’s test. *p* < 0.05 was statistically significant. Western blot band intensities were quantified via ImageJ 1.54p densitometry and normalized to β-actin.

## 5. Conclusions

In summary, this study demonstrates that Mazdutide significantly improves multiple abnormal indicators in high-fat-diet-induced MASLD mice, such as alleviating dyslipidemia, improving liver function, reducing hepatic steatosis, and simultaneously lowering oxidative stress levels and the release of inflammation-related factors. Its hepatocyte-protective effects were further validated in an FFA-induced MASLD cellular model. The mechanism may involve Mazdutide inhibiting endoplasmic reticulum stress (via the PERK-eIF2α-ATF4-CHOP axis) and its associated inflammatory pathways (NF-κB/TNF-α), as well as regulating lipid metabolism-related molecules (SREBP-1, C/EBPβ, PPARγ), thereby improving and delaying MASLD progression. This finding provides new experimental evidence for the molecular mechanism of Mazdutide in improving MASLD and its clinical application in related disease treatment.

## Data Availability

The original contributions presented in this study are included in the article or Supplementary Materials. Further inquiries can be directed to the corresponding authors.

## Supplementary Materials

The following supporting information can be downloaded at https://www.mdpi.com/article/10.3390/ph19030371/s1, Table S1: Macronutrient and energy distribution in normal chow and high-fat diets.

## Abbreviations

The following abbreviations are used in this manuscript:

MASLD	Metabolic dysfunction-associated steatotic liver disease
NAFLD	Non-alcoholic fatty liver disease
MASH	Metabolic dysfunction-associated steatohepatitis
T2DM	Type 2 diabetes mellitus
ER	Endoplasmic reticulum
UPR	Unfolded protein response
PERK	Protein kinase R-like endoplasmic reticulum kinase
eIF2α	Eukaryotic initiation factor 2 alpha
ATF4	Activating transcription factor 4
CHOP	C/EBP homologous protein
GLP-1	Glucagon-like peptide-1
GCG	Glucagon
HFD	High-fat diet
NCD	Normal chow diet
TC	Total cholesterol
TG	Triglycerides
LDL-C	Low-density lipoprotein cholesterol
HDL-C	High-density lipoprotein cholesterol
ALT	Alanine aminotransferase
AST	Aspartate aminotransferase
FFA	Free fatty acid
HE	Hematoxylin and Eosin
TNF-α	Tumor necrosis factor alpha
IL-1β	Interleukin-1 beta
IL-6	Interleukin-6
MDA	Malondialdehyde
SOD	Superoxide dismutase
NF-κB	Nuclear Factor kappa B
SREBP-1	Sterol regulatory element-binding protein 1
C/EBPβ	CCAAT/enhancer-binding protein beta
PPARγ	Peroxisome proliferator-activated receptor gamma
AML-12	Alpha Mouse Liver-12
4-PBA	4-phenylbutyric acid
