# Supplementary material for: Mazdutide Ameliorates Metabolic Dysfunction-Associated Steatotic Liver Disease by Modulating Endoplasmic Reticulum Stress, Improving Lipid Metabolism and Alleviating Inflammation
Source: Pharmaceuticals (Basel). 2026 Feb 26;19(3):371. doi: 10.3390/ph19030371 (PMC13028924; doi:10.3390/ph19030371)
Supplement: Supplementary file 1 [file pharmaceuticals-19-00371-s001.zip › pharmaceuticals-4122367-supplementary.pdf]

Supplementary Materials:

Table S1. Macronutrient and energy distribution in normal chow and high-fat diets

	**NCD**		**HFD**	
	**gm%**	**Kcal%**	**gm%**	**Kcal%**
Carbohydrate	67.1	70	26	20
Proteins	18.9	20	26	20
Fat	4.3	10	35	60
Others	9.7	-	13	-
Total (Kcal/gm)	100 (3.82 Kcal/g)		100 (5.23 Kcal/g)	

Abbreviations: NCD, normal chow diet; HFD, high-fat diet.
